# Therapeutic outcomes of ²²⁵Ac/¹⁷⁷Lu-PSMA combination therapy in advanced metastatic Castration-Resistant prostate cancer: A systematic review and Meta-Analysis

**DOI:** 10.1007/s00259-025-07627-y

**Published:** 2025-11-18

**Authors:** Zineddine Belabaci, Giulio Brignoli, Thomas Zilli, Miloš Grujić, Issa Mohamad, Akram Al-Ibraheem, Mohamed Shelan, Ali Afshar-Oromieh

**Affiliations:** 1 Faculty of Medicine, Djillali Liabes University, Sidi Bel Abbes, Algeria; 2 Department of Radiation Oncology, Inselspital, Bern University Hospital, University of Bern, Bern, Switzerland; 3 Radiation Oncology, Oncology Institute of Southern Switzerland (IOSI), EOC, Bellinzona, Switzerland; 4 Faculty of Biomedical Sciences, Università della Svizzera italiana, Lugano, Switzerland; 5 Faculty of Medicine, University of Geneva, Geneva, Switzerland; 6 Center for Radiation Oncology, University Clinical Centar Kragujevac, Kragujevac, 34000 Serbia; 7 Department of Radiation Oncology, King Hussein Cancer Center, Amman, Jordan; 8 Department of Nuclear Medicine, King Hussein Cancer Center, Amman, Jordan; 9 Department of Nuclear Medicine, Inselspital, Bern University Hospital, University of Bern, Bern, Switzerland

**Keywords:** Metastatic Castration-Resistant prostate cancer (mCRPC), Radionuclide therapy, [225Ac] Ac-/[177Lu] Lu-PSMA, Biochemical response

## Abstract

**Background:**

Targeted radionuclide therapy (TRT) has become a standard of care for patients with metastatic castration-resistant prostate cancer (mCRPC). Lutetium-177 labeled PSMA radioligand therapy ([¹⁷⁷Lu]Lu-PSMA) is an established and effective treatment option, while [²²⁵Ac]Ac-PSMA shows promise in refractory cases. The tandem use of [225Ac] Ac and [177Lu] Lu-labeled PSMA ligands is currently being explored to harness the complementary advantages of both isotopes. This meta-analysis investigates the efficacy and safety of [²²⁵Ac]Ac -/[¹⁷⁷Lu]Lu-PSMA radioligand therapy (RLT) in patients with mCRPC.

**Methods:**

This systematic review is reported in accordance with the Preferred Reporting Items for Systematic Reviews and Meta-Analyses (PRISMA). A comprehensive literature search was performed in PubMed, EMBASE, Web of Science (WOS), and Scopus databases, covering all records from inception through March 2025. The primary endpoints focused on therapeutic efficacy, assessed through PSA-based biochemical responses. These included any PSA decline, a PSA decline of more than 50% from baseline, stable disease (defined as a PSA increase of < 25% PSA increase or a decrease of < 50%), and progressive disease (defined as an increase of ≥ 25% PSA) following tandem 225Ac-/177Lu-PSMA tandem RLT. Secondary endpoints included overall survival (OS) and treatment-related adverse events. A random-effects model was used to generate pooled proportions through meta-analysis.

**Results:**

Eight studies, including a total of 323 patients treated with [²²⁵Ac]-/[¹⁷⁷Lu]-PSMA combination therapy, were analyzed. The pooled response rates showed that 47% (95% CI: 37%–56%) experienced a PSA decline greater than 50%, while 78% (95% CI: 70%–86%) had any measurable PSA decline. The estimated median OS was 11.8 months (95% CI: 9.0–14.6 months). Severe toxicities were infrequent; the most common severe grade ≥ 3 adverse events were anemia (10%) and thrombocytopenia (6%). No cases of grade ≥ 3 xerostomia were reported.

**Conclusion:**

[²²⁵Ac]Ac-/[¹⁷⁷Lu]Lu-PSMA RLT shows encouraging activity and manageable safety in patients with advanced mCRPC. Given the retrospective nature of the available evidence and limited data, these findings should be further evaluated in prospective trials to determine long-term efficacy and survival outcomes.

**Supplementary Information:**

The online version contains supplementary material available at 10.1007/s00259-025-07627-y.

## Introduction

Following a prostate cancer diagnosis, 10–20% of patients progress to castration-resistant prostate cancer (CRPC), with most (>80%) presenting with advanced or metastatic disease (mCRPC) [1].Standard therapies, such as androgen receptor pathway inhibitors (ARPI), taxane-based chemotherapy, and Radium-223, provide limited survival benefits, typically extending median survival by 3–4 months per treatment line [2, 3].

Radiopharmaceuticals have become valuable tools in cancer care, enabling both diagnosis and treatment. The development of theranostics, using radiolabeled compounds for patient selection, dosing, and response monitoring, has advanced the field of targeted radionuclide therapy (TRT) [4, 5].Both α- and β-emitting radionuclides are used therapeutically, with Lutetium-177 emerging as a widely used β-emitter due to its favorable properties and availability. It has shown promise in treating advanced, unresectable, or metastatic cancers [6].

[¹⁷⁷Lu]Lu-PSMA-617 is a Food and Drug Administration (FDA)-approved treatment for mCRPC in patients previously treated with ARPI. Its efficacy was demonstrated in the VISION, TheraP, and PSMAfore trials [7–9]. The VISION trial reported a median overall survival of 15.3 months and a radiographic progression-free survival of 8.7 months with [¹⁷⁷Lu]Lu-PSMA-617 therapy, and a PSA50 response rate (≥ 50% PSA decline) of approximately 46%. However, Up to 30% of patients do not respond, and most responders eventually experience disease progression. The mechanisms behind primary and acquired resistance remain unclear. To overcome this, alternative PSMA RLT agents can be employed, including [¹⁶¹Tb]Tb-PSMA and [²²⁵Ac]Ac-PSMA in the form of monotherapy or combination therapy approaches [10–12].

Among all α-emitting radionuclides, Actinium-225 is considered the most suitable for targeted alpha therapy due to its optimal half-life, potent cytotoxicity, and short tissue penetration which enables a more precise treatment. [²²⁵Ac]Ac-PSMA-617 has shown promising results in mCRPC patients who progressed after [¹⁷⁷Lu]Lu-PSMA-617 [13]. However, xerostomia is a common side effect [14]. To improve tolerability without compromising therapeutic efficacy, tandem therapy using lower Actinium-225 activity combined with [¹⁷⁷Lu]Lu-PSMA-617 has been explored [15, 16].

This meta-analysis evaluates the efficacy and safety of combined [²²⁵Ac]/[¹⁷⁷Lu] PSMA RLT in patients with mCRPC.

## Methods

This systematic review and meta-analysis is reported in line with the PRISMA 2020 statement [17–20] and is registered in PROSPERO (CRD42025644048). 

### Search strategy and study selection

A comprehensive literature search was conducted in March 2025 using the PubMed/MEDLINE, EMBASE, Web of Science, and Scopus databases, with terms related to PSMA-targeted radionuclide therapy and prostate cancer. The search combined keywords such as (“Lutetium” OR “Lu” OR “Actinium” OR “Ac”) AND (“combination” OR “tandem” OR “cocktail” OR “augmented”) AND (“Prostate-specific membrane antigen” OR “PSMA”) AND (“Prostate cancer” OR “Prostate neoplasm” OR “mCRPC” OR related terms. Publication bias was evaluated by visual inspection of funnel plots and by Egger’s linear regression test for funnel plot asymmetry across all outcomes.

In addition, the reference lists of all included articles were screened to identify any further eligible studies.

After removing duplicates, two authors (ZB and MS) independently screened titles and abstracts using Rayyan (Rayyan Systems, Cambridge, MA, USA), followed by full-text review. Disagreements were resolved by discussion or third-party adjudication. Studies were included if they enrolled patients with mCRPC and reported combination therapy with Actinium-225 and Lutetium-177 PSMA-targeted agents, including complete data on biochemical response (≥ 50% PSA decline, progressive disease, and stable disease). Exclusion criteria included non-human studies, reviews, editorials, case reports, non-English articles, studies on non-mCRPC populations, and those focused only on biodistribution or dosimetry.

### Data extraction and outcomes

Two reviewers (ZB and GB) independently extracted data from included studies. Variables included author, year, study design, country, patient age, baseline PSA, sample size, metastatic burden, type of radioligands, number of cycles, administered activity per cycle (GBq), and prior exposure to [¹⁷⁷Lu]Lu-PSMA or [²²⁵Ac]Ac-PSMA monotherapy. Primary outcomes were biochemical responses: any PSA decline, > 50% PSA decline, stable disease (< 25% PSA increase or < 50% decrease), and progressive disease (≥ 25% PSA increase). Secondary outcomes included overall survival (OS) and PSA progression-free survival (PFS), with medians and 95% Confidence Intervals (CI) extracted. Safety outcomes included grade 1–2 and grade 3–5 adverse events per CTCAE criteria.

### Quality assessment

Study quality was assessed using the Newcastle-Ottawa Scale for observational studies (NOS) [21–25], which evaluates selection, comparability, and outcomes. Scores ≥ 7 were considered high quality, and scores < 5 were classified as low quality. Two reviewers independently assessed quality, resolving disagreements by discussion.

### Statistical analysis

Statistical analysis was conducted using R 4.5.0 and RStudio software, employing the meta package (metaprop) for proportions and the metamedian package for median overall survival. Pooled event rates and 95% CI were calculated using a random-effects model with the restricted maximum-likelihood (REML) estimator. Forest plots were generated to visualize pooled proportions for PSA response. Heterogeneity was assessed using Cochran’s Q test and the I² statistic, with thresholds of 25%, 50%, and 75% indicating low, moderate, and high heterogeneity, respectively. Publication bias was evaluated using Egger’s test. Statistical significance was set at *p* < 0.05.

## Results

### Study selection

**Fig. 1:**
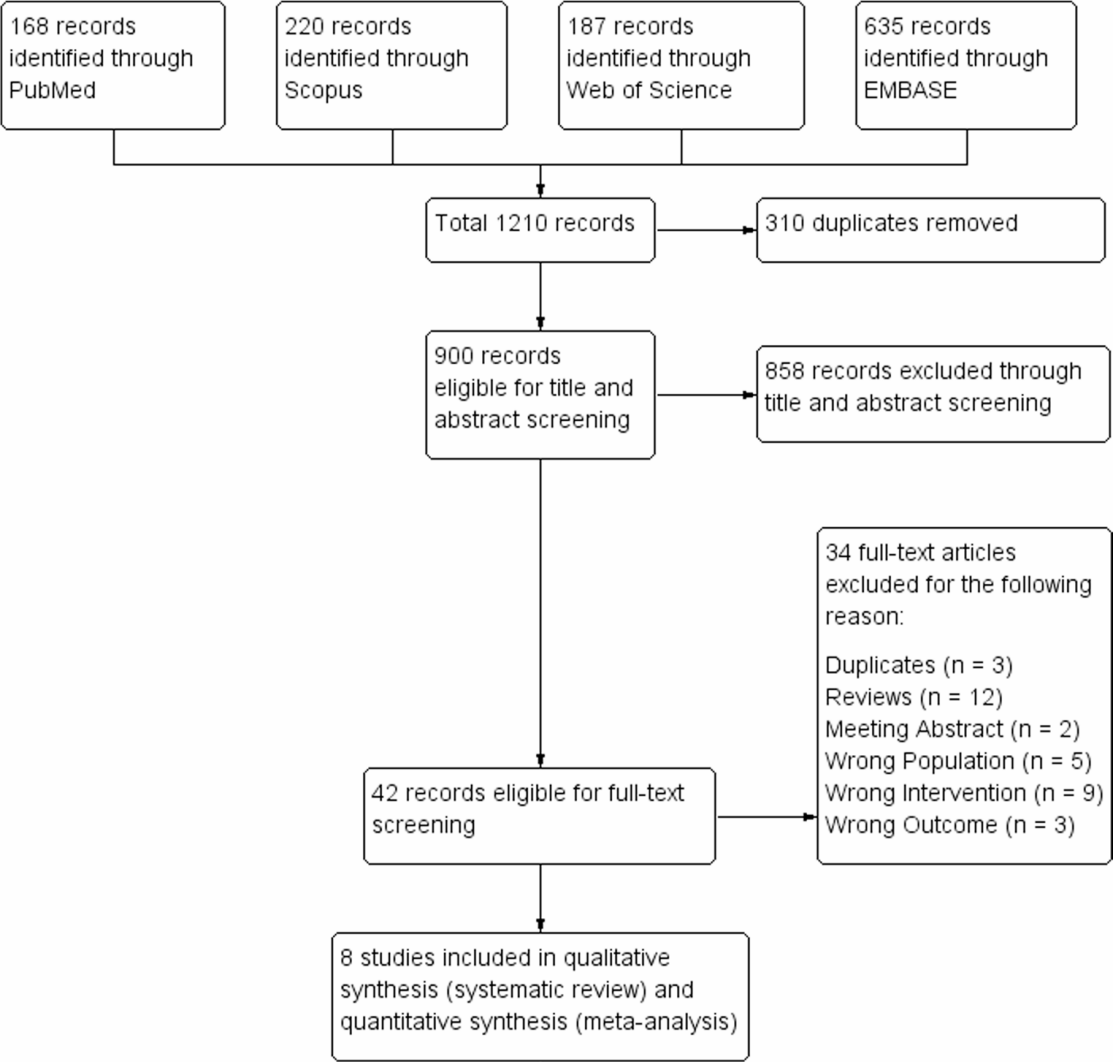
PRISMA flow diagram of the study selection process

The initial search identified 1,210 records. After removing 310 duplicates, 900 titles and abstracts were screened. Forty-two articles underwent full-text review, and 8 retrospective studies from Germany met the inclusion criteria [26–33]. The study selection process is shown in the PRISMA flow diagram **(**Fig. 1**)**.

### Study characteristics

A total of 323 mCRPC patients received [²²⁵Ac]Ac-/[¹⁷⁷Lu]Lu-PSMA tandem therapy. Median patient age ranged from 62 to 77 years, and baseline PSA levels ranged from 78 to 416 ng/mL. All studies included patients who received at least one cycle of tandem therapy with a mean of approximately 2 ± 1 cycles, though treatment regimens differed in the amount of activity administered; the mean per-cycle activity was approximately 3.8 MBq for [²²⁵Ac]Ac-PSMA and 6.0 GBq for [¹⁷⁷Lu]Lu-PSMA **(**Table 1**)**. Across the included studies, prior exposure to [¹⁷⁷Lu]Lu-PSMA therapy varied. Four studies included patients previously treated with [¹⁷⁷Lu]Lu-PSMA [27, 30, 32, 33], while others either excluded such patients [29, 31] or included mixed populations [26, 28]. Metastatic burden (bone, lymph node, visceral) was variably reported. Detailed study characteristics are summarized in Table 1.

**Table 1:** Baseline characteristics and main outcomes of the included studies

Study	Patients (*n*)	Ac-PSMA Agent	Lu-PSMA Agent	Age (median, range)	Baseline PSA level (ng/mL) (median, range)	Extent of Metastasis (%)	Previous LuPSMA (%)	Number of cycles Median (range)		Activity per cycle Median (range)		Main Outcomes
								Ac-PSMA	Lu-PSMA	Ac-PSMA	Lu-PSMA	
Sheikh et al. 2025	25	Ac-PSMA-I&T	Lu-PSMA-I&T	75 (60–87)	132 (0.03–1512)	bone 100% lymph nodes 88% liver 12% lung 8% peritoneum 8%	177Lu-PSMA RLT 32% 225Ac-PSMA TAT 8%	2 (1–8)	2 (1–8)	5.97 (1.92– 8.70 MBq)	1073 (559–4328 MBq)	PSA50 : 48% Any PSA decline : 68% mOS :11.86 months, CI [7.0–16.7.0.7] mPFS: NA
Burgard al. 2024	33	Ac-PSMA-617	Lu-PSMA-617	71 (58–85)	98 (8–2307)	Bone 91% Lymph node 56% Liver 26% Lung 9%	100%	mean of 2 ± 1 augmentations	a mean of 2 ± 1 cycles	mean of 3.8 ± 1.7 MBq	mean of 5.4 ± 1.7 GBq	PSA50 : 24% Any PSA decline : NA mOS :7 months, CI [4–11] mPFS: NA
Rathke et al. 2024	233 (129 Ac-/Lu- tandem therapy; 104 Ac-PSMA montherapy)	Ac-PSMA-617	Lu-PSMA-617	Ac-/Lu-PSMA 62 (44–80)	78 (0–6557)	Lung 8% Liver 15% Lymph node 67% Bone 97% Bone marrow (superscan) 39% Brain 1% Other 15%	32%	the intention-to-treat protocol was a 3-cycle therapy	the intention-to-treat protocol was a 3-cycle therapy	4 MBq (average of 4.3 MBq/cycle)	4 GBq	PSA50 : 57% Any PSA decline : NA mOS : 15 months, CI [11–19] mPFS: NA
				Ac-PSMA 62 (48–81)	312 (0–4,843)	Lung 13% Liver 22% Lymph node 70% Bone 96% Bone marrow (superscan) 76% Brain 2% Other 21%	37%	the intention-to-treat protocol was a 3-cycle therapy	-	(average of 6.6 MBq/cycle)	-	PSA50 : 53% Any PSA decline : NA mOS : 9 months, CI [7.2–10.8] mPFS: NA
Rosar et al. 2024	33	Ac-PSMA-617	Lu-PSMA-617	75 (55–89)	416 (1–5683)	Bone 97% Lymph node 73% Liver 28% Lung 18% Other 36%	0	1–2 cycles	2 cycles	mean 2.6 ± 1.6 MBq (range: 0.5–9.2 MBq)	mean 6.9 ± 1.7 GBq (range: 1.3–10.5 GBq)	PSA50 : 48% Any PSA decline : 79% mOS : 14.8 months, CI [8.3–21.3] mPFS:7.2 months, CI [5.9–8.5]
Rosar et al. 2024	51	Ac-PSMA-617	Lu-PSMA-617	72.5 (51–97)	191 (6–5547)	Bone 96.1% Lymph node 66.7% Liver 15.7% Lung 7.8% Other 19.6%	100%	mean of 2 ± 1 (1–6)	mean of 2 ± 2 (1–8)	mean of 3.9 ± 1.7 MBq	mean of 6.0 ± 1.4 GBq	PSA50 : 47% Any PSA decline : 71% mOS : 9.1 months, CI [5.4–12.8] mPFS:6.3 months, CI [3.9–10]
Rosar et al. 2021	15	Ac-PSMA-617	Lu-PSMA-617	77 (57–88)	272 (58–3389)	Bone 100% Lymph node 73% Liver 40% Lung 13% Other 7%	0	1–2	All patients received 2 cycles	mean administered activity of augmentation per cycle was 2.7 ± 1.1 MBq	mean administered activity 6.7 ± 1.8 GBq	PSA50 : 53% Any PSA decline : 93% mOS : 14.8 months, CI [9.6–16.9] mPFS: 9.1 months, CI [3.7–10.4]
Rosar et al. 2021	17	Ac-PSMA-617	Lu-PSMA-617	69.4 ± 8.3 (57.0–89.0)	152 (5.9–2570)	Bone 100% Lymph node 29.4% Liver 11.8% Other 5.9%	100%	1 cycle tandem	1 cycle tandem	4 ± 2MBq (range: 1.8–6.9)	6.0 ± 1 GBq (range: 3.8–8.2 GBq)	PSA50 : 29% Any PSA decline : 59% mOS: PR vs. SD/PD by imaging: not reached vs. 8.3 months mPFS: 3.7 months, CI [3–4.4.4]
Khreish et al. 2020	20	Ac-PSMA-617	Lu-PSMA-617	72 (57–88)	215 (6–5547)	Bone 95% Lymph node 45% Liver 20% Lung 10%	100%	1 cycle	1 Cycle as tandem; 1 (0–5) cycles of post-tandem maintenance 177Lu-PSMA-617 monotherapy	5.3 (1.5–7.9) MBq	6.9 (5.0–11.6) GBq	PSA50 : 65% Any PSA decline : 90% mOS : 48 weeks, CI [4–9] mPFS: 19 weeks, CI [12–26]

Abbreviations: *PSA* prostate specific antigen, PSA50: ≥50% decline in PSA value from baseline, *CI* 95% confidence interval, *mOS* median overall survival, *mPFS* median progression-freesurvival, *NA* not available

### Quality assessment

All studies were rated as high quality according to the Newcastle-Ottawa Scale score for observational studies. Details are available in Table 2. Funnel plot inspection suggested asymmetry for SD, while no clear asymmetry was observed for PSA50, PD, or any PSA decline. Egger’s test confirmed small-study effects for SD (*p* = 0.02), whereas results were not significant for PSA50 (*p* = 0.45), PD (*p* = 0.10), or any PSA decline (*p* = 0.82). Analyses of publication bias are presented in the Supplement (Figure S1A–D), where funnel plots and Egger’s tests are reported for all outcomes.

**Table 2:** Newcastle–Ottawa scale quality assessment of the included studies

Study	Selection	Comparability	Outcome	Score
Sheikh et al. 2025	**3**	**2**	**2**	**7**
Burgard al. 2024	**3**	**2**	**2**	**7**
Rathke et al. 2024	**4**	**2**	**3**	**9**
Rosar et al. 2024	**3**	**2**	**3**	**8**
Rosar et al. 2024	**3**	**2**	**3**	**8**
Rosar et al. 2021	**3**	**2**	**2**	**7**
Rosar et al. 2021	**3**	**2**	**3**	**8**
Khreish et al. 2020	**3**	**1**	**3**	**7**

### Efficacy of Ac-/Lu-PSMA RLT

**Fig. 2:**
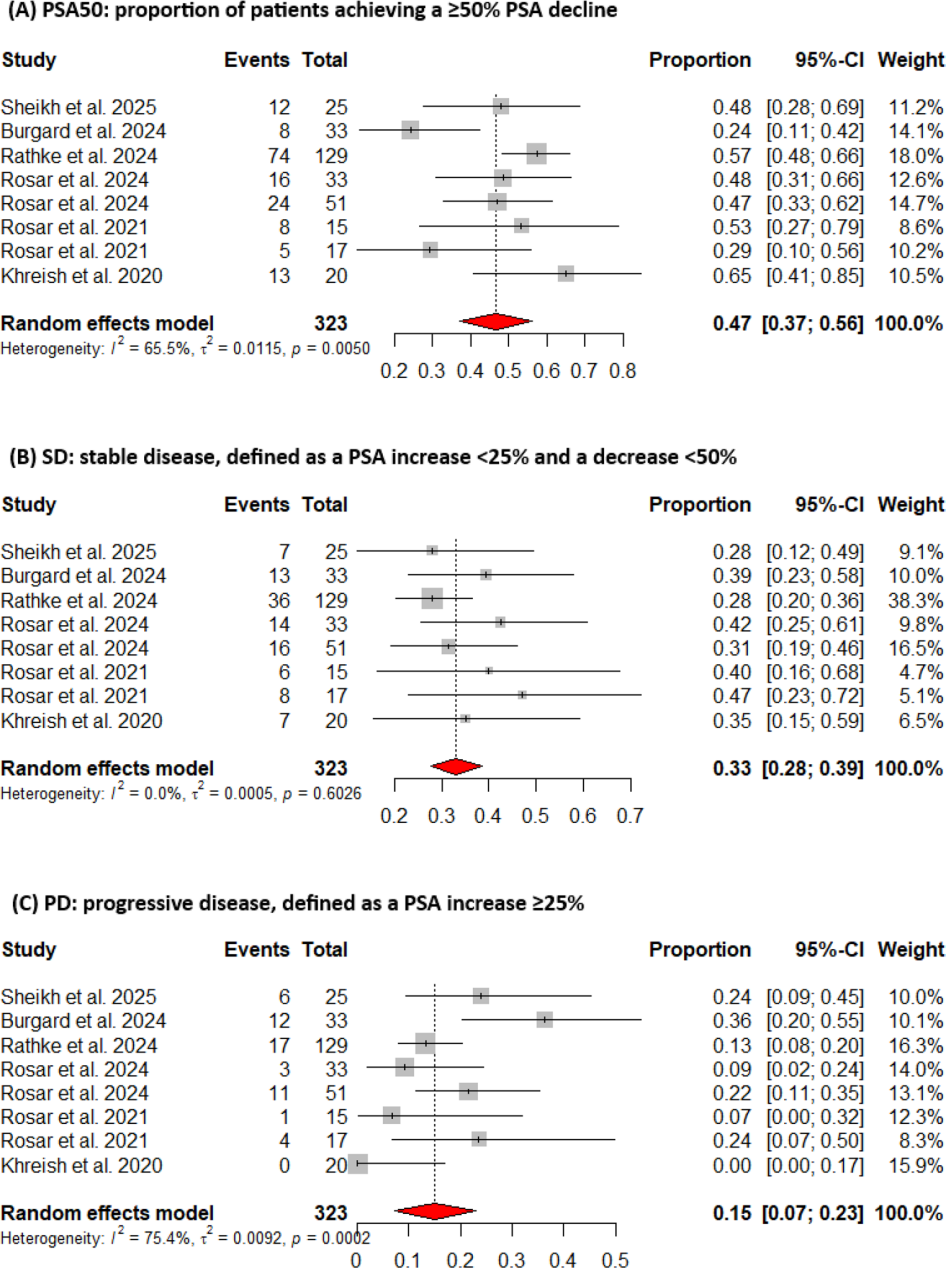
Forest plots of pooled proportions of PSA responses. (**A**) Proportion of patients achieving PSA50 response. (**B**) Proportion of patients with stable disease (SD). (**C**) Proportion of patients with progressive disease (PD). Squares represent study-specific estimates with sizes proportional to weight, horizontal lines indicate 95% confidence intervals, and diamonds show pooled random-effects estimates

**Fig. 3:**
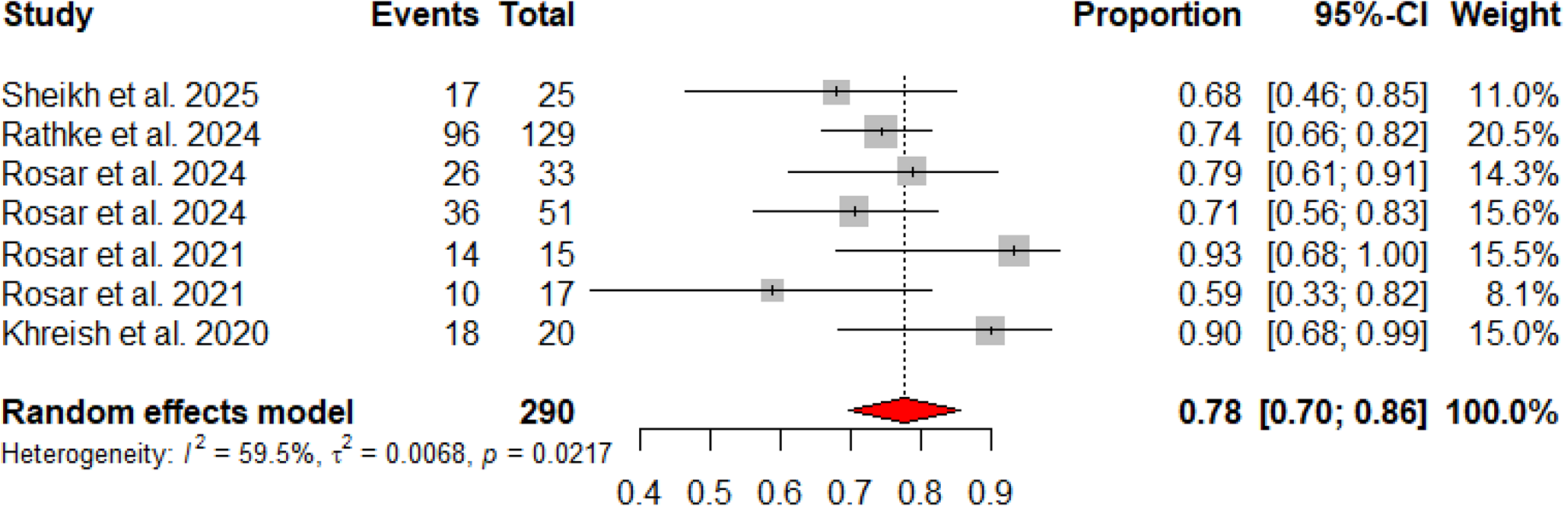
Forest plot of the proportion of patients with any PSA decline

All studies reported biochemical response categorizing outcomes as: response (≥ 50% decline), stable disease (− 50% to + 25% change), or progressive disease (≥ 25% increase). All studies provided OS data, and five reported PSA progression-free survival (PSA-PFS) **(**Table 1**)**. The pooled rates of patients achieving a PSA decline ≥ 50% and any PSA decline were 47% (95% CI: 37%–56%) and 78% (95% CI: 70%–86%), respectively, with moderate heterogeneity (I² = 65.5%, *p* = 0.005 and I² = 59.5%, *p* = 0.021) (Figs. 2A and 3) This variability may reflect differences in patient populations, treatment regimens, or prior therapies.

**Fig. 4:**
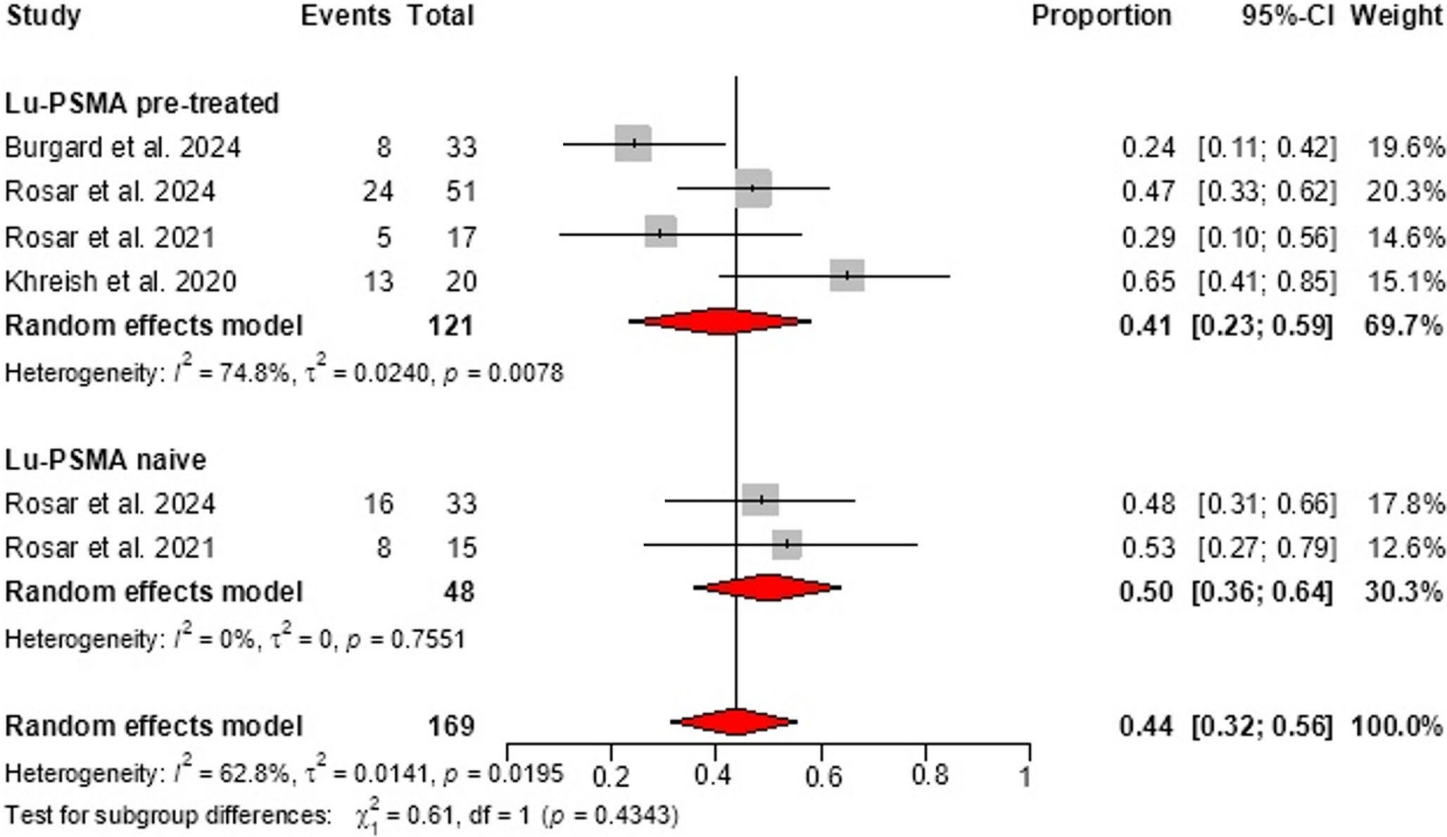
Subgroup analysis of PSA50 response according to prior exposure to [177Lu]Lu-PSMA. The lutetium-naïve subgroup included two studies, which limits the ability to estimate heterogeneity. Pooled proportions are displayed as diamonds, with horizontal lines representing 95% confidence intervals

A subgroup analysis of six studies [27, 29–33] (169 patients) compared those previously treated with 177Lu-PSMA to naive patients. The PSA ≥ 50% response was 41% (95% CI: 23%–59%) in the pre-treated group with high heterogeneity (I² = 74.8%, *p* = 0.018), and 50% (95% CI: 36%–64%) in the naive group with no heterogeneity (I² = 0%). The difference between subgroups was not statistically significant (*p* = 0.434). Overall, the pooled response rate was 44% (95% CI: 32%–56%) with moderate heterogeneity (I² = 62.8%, *p* = 0.020) **(**Fig. 4**)**. The pooled rates of stable disease and progressive disease were 33% (95% CI: 28%–39%) and 15% (95% CI: 7%–23%), respectively **(**Fig. 2B and C**)**. The pooled estimate of median OS was 11.8 months (95% CI: 9.0–14.6) with moderate heterogeneity (I² = 60%, *p* = 0.02). Five studies reported PFS medians ranging from 3.7 to 9.1 months **(**Table 1**)**.

### Safety and adverse events

Adverse events were mostly mild to moderate (grade 1–2). The most common severe events (grade ≥ 3) were anemia (10%, 14/136 patients) and thrombocytopenia (6%, 9/136). Moreover, grade ≥ 3 anemia was observed in 12% of [¹⁷⁷Lu]Lu-PSMA pre-treated patients and 6% of [¹⁷⁷Lu]Lu-PSMA naive patients. No cases of xerostomia greater than grade 2 were reported. Details by type and severity are in Table 3.

**Table 3:** Overview of adverse events reported with [225Ac]Ac-/[177Lu]Lu-PSMA combination therapy

Event	Grade 1–2 *n*(%)	Grade ≥ 3 *n*(%)
Anemia	79/116 (68%)	14/136 (10%)
Leukopenia	35/116 (30%)	3/136 (2%)
Thrombocytopenia	31/116 (26%)	9/136 (6%)
Nephrotoxicity	53/116 (46%)	1/136 (0.7%)
Xerostomia	189/265 (71%)	0/265 (0%)

## Discussion

mCRPC remains a therapeutic challenge, particularly in advanced stages where resistance to standard treatments is common and few options are available. Therefore, new therapeutic approaches are needed to achieve better outcomes for patients, particularly in late-stage/end-stage prostate cancer.

Feuerecker et al. reported that switching to [²²⁵Ac]Ac-PSMA-617 monotherapy after failure of [¹⁷⁷Lu]Lu-PSMA RLT offers an effective treatment option [34]. However, [²²⁵Ac]Ac-PSMA monotherapy appears to result in a higher incidence of adverse events [34–37]. In a recent international multicenter study involving 488 patients treated with [²²⁵Ac]Ac-PSMA RLT, Sathekge et al. reported xerostomia in 68% after the first cycle of [²²⁵Ac]Ac-PSMA monotherapy, increasing with additional treatment cycles [35].

Our meta-analysis represents the first comprehensive synthesis of available data on the efficacy and safety of [²²⁵Ac]Ac-/[¹⁷⁷Lu]Lu-PSMA combination RLT in patients with mCRPC, addressing a novel and potential option for mCRPC. The assessment showed that 47% of patients achieved a PSA reduction of 50% or more following Ac-/Lu-PSMA tandem RLT. Although lower than the response rates reported by Dai et al. [38]for [¹⁷⁷Lu]Lu-PSMA RLT (49%) and [²²⁵Ac]Ac-PSMA (60%), the Ac/Lu-PSMA RLT combination approach was commonly used in advanced mCRPC patients who are heavily pre-treated, and may be refractory to [¹⁷⁷Lu]Lu-PSMA RLT [27, 30, 32, 33]. Moreover, the estimated proportion of patients with any PSA decline was 78%, highlighting the potential benefit of the Ac-/Lu-PSMA tandem approach, even in a population that is heavily pre-treated. These values are comparable to those observed in the VISION trial [7], which reported a PSA50 response rate of 46% and any PSA decline in approximately 66% of patients.

The pooled median overall survival was 11.8 months, shorter than the 15.3 months reported in the VISION trial, likely reflecting the more heavily pre-treated population included in the tandem therapy studies. This survival duration is consistent with what might be expected in later-line settings, where therapeutic options are limited. However, long-term follow-up and PFS data were scarce or inconsistent, with median ranging from 3.7 to 9.1 months across studies. This range overlaps with the median PFS of 8.7 months reported in the VISION trial. The pooled mOS represents a useful summary but should be interpreted in light of inter-study variability and possible deviations from the actual effect size.

The safety profile of the Ac-/Lu-PSMA tandem therapy was generally favorable, with most adverse events being mild to moderate. These findings are largely consistent with the meta-analysis by Ninatti et al. [13] on α-RLT, though the incidence of adverse events in our analysis was slightly lower. Notably, fewer CTCAE grade 1–2 cases and no grade ≥ 3 xerostomia were reported, suggesting the tandem approach may reduce salivary gland toxicity commonly seen with [²²⁵Ac]Ac-PSMA monotherapy. In contrast, the VISION trial reported grade ≥ 3 adverse events in 52.7% of patients, suggesting a more favorable toxicity profile for the tandem approach. However, given the small patient population, the low number of administered tandem cycles, and the absence of long-term follow-up data, these findings should be interpreted with caution. Across studies, patients received on average only two tandem RLT cycles, leaving it unclear whether additional cycles would increase the risk of toxicity, including grade ≥ 3 xerostomia.

Extended use and rechallenge of [¹⁷⁷Lu]Lu-PSMA RLT have shown promising clinical feasibility and tolerability. Seifert et al. [39], indicate that administering more than six cycles of [¹⁷⁷Lu]Lu-PSMA-617 is generally well tolerated, without signs of cumulative toxicity. This supports the concept that selected patients may benefit from continued or repeated treatment beyond standard protocols. In particular, rechallenge with [¹⁷⁷Lu]Lu-PSMA after initial response and subsequent progression has been explored as a viable strategy. Retreatment was associated with renewed PSA responses and manageable safety profiles in selected patients despite earlier exposure [39–42]. These findings open the door to more flexible, patient-adapted approaches, especially in settings where other treatment options are exhausted or poorly tolerated. Furthermore, combining [¹⁷⁷Lu]Lu-PSMA with [²²⁵Ac]Ac-PSMA, either sequentially or in tandem, has shown early signals of benefit without introducing major new safety concerns. Despite these encouraging results, most available data are from retrospective series with limited sample sizes from the same region, with about half of the included patients treated in the same department by Rosar et al. Further data from different populations would be important to strengthen the evidence base.

This meta-analysis has several limitations. There was heterogeneity across studies in terms of patient populations, treatment protocols, administered activities, number of cycles, dosing intervals, and baseline clinical characteristics, including prior lines of therapy, presence of visceral metastases, and functional status. These differences complicate direct comparisons and affect the reliability of pooled estimates. Furthermore, all studies were retrospective and lacked control groups, introducing selection bias and limiting the strength of the evidence. The absence of randomized controlled trials means that findings rely on observational data and may be subject to confounding. Additionally, small sample sizes and limited follow-up in many studies restrict conclusions about long-term outcomes such as survival and delayed toxicities. Despite these limitations, this work highlights the clinical potential of the Ac-/Lu-PSMA combination approach as a promising therapeutic option for patients with advanced mCRPC, particularly in settings where standard treatments have failed.

## Conclusion

The findings of this meta-analysis support the potential role of [²²⁵Ac]Ac-/[¹⁷⁷Lu]Lu-PSMA combination therapy as a treatment option for patients with advanced mCRPC. The approach demonstrated meaningful efficacy and a manageable safety profile, with a lower incidence of certain toxicities compared to lutetium or actinium monotherapy. While current evidence is limited by study heterogeneity and the lack of prospective data, the results justify further clinical investigation. Well-designed randomized trials are needed to confirm these outcomes, define optimal treatment protocols, and determine the place of tandem therapy in the evolving landscape of prostate cancer management.

## Supplementary Information

Below is the link to the electronic supplementary material.

Supplementary Material 1 (DOCX 52.3 KB)

## Data Availability

All the data have been presented here in the manuscript; any additional data can be provided by contacting the corresponding author.
