# Supplementary material for: Therapeutic outcomes of ²²⁵Ac/¹⁷⁷Lu-PSMA combination therapy in advanced metastatic Castration-Resistant prostate cancer: A systematic review and Meta-Analysis
Source: Eur J Nucl Med Mol Imaging. 2025 Nov 18;53(5):2881–92. doi: 10.1007/s00259-025-07627-y (PMC13013121; doi:10.1007/s00259-025-07627-y)
Supplement: Supplementary file 1 — Supplementary Material 1 (DOCX 52.3 KB) [file 259_2025_7627_MOESM1_ESM.docx]

**PICO Framework ( Eligibility Criteria )**

**Title**

**Therapeutic Outcomes of [225Ac]Ac-/[177Lu]Lu-PSMA Combination Therapy in Advanced Metastatic Castration-Resistant Prostate Cancer: A Systematic Review and Meta-Analysis**

- **P (Population)**: Adult Men with a diagnosis of metastatic castration-resistant prostate cancer
- **I (Intervention)**: [225Ac]Ac-/[177Lu]Lu- PSMA combination therapy
- **C (Comparator):** [225Ac]Ac-PSMA monotherapy OR /[177Lu]Lu- PSMA monotherapy OR no comparator (single arm studies)
- **O (Outcome)**: primary : PSA responses.

Secondary : treatment-related adverse events (AE), Overall survival(OS), progression-free survival(PFS)

**Inclusion Criteria** :

Adult men (aged >18 years) with a diagnosis of Metastatic Castration-Resistant Prostate Cancer.

Randomized clinical trials, prospective studies, and retrospective studies that established the efficacy and safety of [225Ac]Ac-/[177Lu]Lu- PSMA combination therapy.

**Exclusion Criteria**:

Basic science articles, non-human subjects, guidelines, case reports, editorials, replies, commentary, reviews, non- English language articles, non-prostate cancer articles, and metastatic castrate sensitive prostate cancer articles to be excluded.

**Funnel Plots for Assessment of Publication Bias in Prostate-Specific Antigen (PSA) Response**


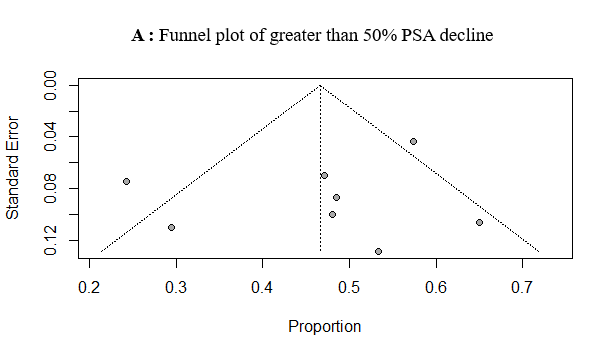

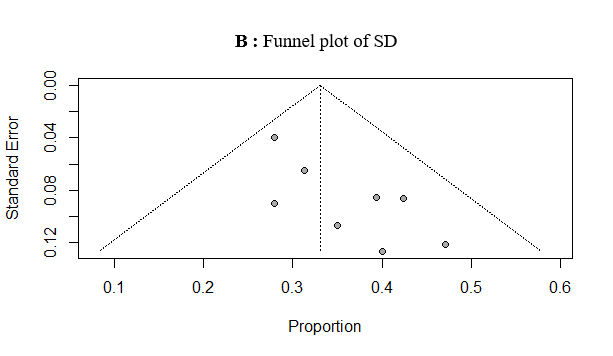


**
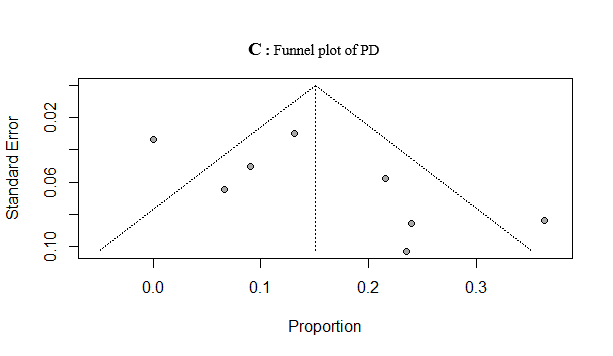

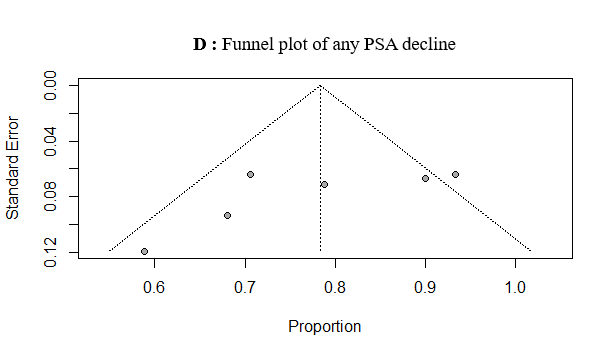
**

**Figure S1.** Funnel plots for Assessment of Publication Bias. (A) PSA50 response, (B) stable disease (SD), (C) progressive disease (PD), and (D) any PSA decline.

**Assessment of publication bias**
Funnel plots and Egger’s linear regression tests were used to explore potential small-study effects. For PSA50, the funnel plot did not indicate clear asymmetry, and Egger’s test was non-significant (t = –0.81, df = 6, p = 0.45). For SD, the funnel plot suggested asymmetry, which was confirmed by Egger’s test (t = 3.12, df = 6, p = 0.02). For PD, visual inspection showed no clear asymmetry, and Egger’s test was not significant (t = 1.94, df = 6, p = 0.10). For any PSA decline, both visual inspection and Egger’s test were negative (t = –0.24, df = 5, p = 0.82). Overall, these findings suggest potential publication bias in SD, but not in the other outcomes.
